# Developmental dynamics of butterfly wings: real-time *in vivo* whole-wing imaging of twelve butterfly species

**DOI:** 10.1038/s41598-018-34990-8

**Published:** 2018-11-15

**Authors:** Masaki Iwata, Motosuke Tsutsumi, Joji M. Otaki

**Affiliations:** The BCPH Unit of Molecular Physiology, Department of Chemistry, Biology and Marine Science, University of the Ryukyus, Okinawa, 903-0213 Japan

## Abstract

Colour pattern development of butterfly wings has been studied from several different approaches. However, developmental changes in the pupal wing tissues have rarely been documented visually. In this study, we recorded real-time developmental changes of the pupal whole wings of 9 nymphalid, 2 lycaenid, and 1 pierid species *in vivo*, from immediately after pupation to eclosion, using the forewing-lift method. The developmental period was roughly divided into four sequential stages. At the very early stage, the wing tissue was transparent, but at the second stage, it became semi-transparent and showed dynamic peripheral adjustment and slow low-frequency contractions. At this stage, the wing peripheral portion diminished in size, but simultaneously, the ventral epithelium expanded in size. Likely because of scale growth, the wing tissue became deeply whitish at the second and third stages, followed by pigment deposition and structural colour expression at the fourth stage. Some red or yellow (light-colour) areas that emerged early were “overpainted” by expanding black areas, suggesting the coexistence of two morphogenic signals in some scale cells. The discal spot emerged first in some nymphalid species, as though it organised the entire development of colour patterns. These results indicated the dynamic wing developmental processes common in butterflies.

## Introduction

Diverse colour patterns of butterfly wings have attracted many artists, amateur lepidopterists, and biologists worldwide throughout the history. No matter how diverse, butterfly wing colour patterns are believed to be constructed based on a common scheme called the nymphalid groundplan^1–5^. The nymphalid groundplan contains three major symmetry systems (the central, border, and basal symmetry systems) and two peripheral systems (the marginal and wing-root systems). Each system is a collection of colour-pattern elements, a core element and a pair of paracore elements at both sides of the core element^3^. Among them, the border symmetry system is probably the most conspicuous in many butterflies because the border ocelli (i.e., eyespots) contain serial concentrating rings of different colours.

Classically, mechanisms of butterfly wing colour pattern formation have been studied using surgical^6–13^, physiological^14–19^, morphological (including colour pattern analysis)^20–27^, and developmental methods^28,29^. These studies contributed to understanding how butterfly wing colour patterns are determined. For example, damage and transplantation experiments demonstrated that the presumptive eyespot centre functions as the organiser for the eyespot^1,6–11^. Pupal cuticle spots have been identified to show the location of organisers for important colour-pattern elements^20,25^. Scale position and scale size are correlated^21^, and scale size and scale colour are also correlated^5,26^, suggesting that the morphogenic signals for colour determination may also determine scale size and hence scale cell size^28^. This discovery has led to the formulation of the ploidy hypothesis^5,26^. Focal white areas in an eyespot of *Calisto* butterflies are clearly independent of the rest of the eyespot, suggesting uncoupling behaviour of the sub-elements within an element^5,27^. A sequence of pigment deposition on the wings was observed at various time points in 1980 using multiple specimens of *Junonia coenia*^1,29^; reddish pigment emerges before black pigment. This process may be called the light-to-dark rule. This time difference has been explained by the difference of their biosynthetic pathways^1,29^.

The colour pattern formation process has been investigated in several different butterflies at the molecular level. Early molecular attempts have focused on gene expression patterns without functional evidence^30–33^. However, functional assays have been developed including gene transfer^34–36^, RNAi knockdown^37–39^, and CRISPR/Cas9 genome editing knockout^40–43^. Furthermore, next-generation genome or RNA sequencing studies have facilitated the identification of potential genes for colour pattern formation^40,44–46^. These studies revealed important functions of some genes involved in the colour pattern formation of eyespots and other elements.

As reviewed in Beldade and Peralta (2017)^47^, a different but equally important approach in butterfly biology is to employ visualisation techniques for live developing pupal wings *in vivo*^48–51^. We have performed live cell *in vivo* imaging with the forewing-lift method using the blue pansy butterfly *Junonia orithya*, which was presented first in Kusaba and Otaki (2009)^21^ and in subsequent studies^48,49^. Iwata *et al*.^48^ have reported colour pattern sequence, secondary tracheal development, scale alignment, haemocyte movement, and contraction pulses. Subsequently, Ohno and Otaki (2015)^49^ investigated the cellular makings of the wing epithelium. Importantly, long-range slow calcium waves are detected on the developing pupal wings^50^. Recently, eyespot organisers were directly observed *in vivo* for the first time at the cellular level^51^.

However, more detailed analyses on the colour pattern sequence and tissue changes are expected to reveal potentially important features in butterfly wing colour pattern development. Moreover, thus far, the imaging data are available only from one species, *J. orithya*, in the literature^21,48–51^. Analyses of different species are necessary to examine the generality of the previous discoveries and to search for additional phenomena that have not been discovered yet.

Here, we examined the pupal wing development of 9 nymphalid butterfly species at the tissue level that covered four subfamilies (Nymphalinae, Heliconiinae, Limenitidinae, and Danainae) available in Okinawa, Japan. These 9 species have unique colour pattern features that represent different aspects of the nymphalid butterflies (see Methods). Additionally, 2 lycaenid butterflies and 1 pierid butterfly were analysed. In most cases, immediately after pupation, the forewing was lifted, and the exposed wing surfaces were monitored by recording the whole-wing images every minute. Thus, image numbers (Panel numbers in figures) roughly correspond to post-pupation time in minute. For transparent or semi-transparent pupae, the wing pattern development was monitored without the forewing lift. This series of simple imaging data revealed developmental facts important to understanding wing development in butterflies.

## Results

### Hindwing and forewing development of *Junonia almana*

**Figure 1:**
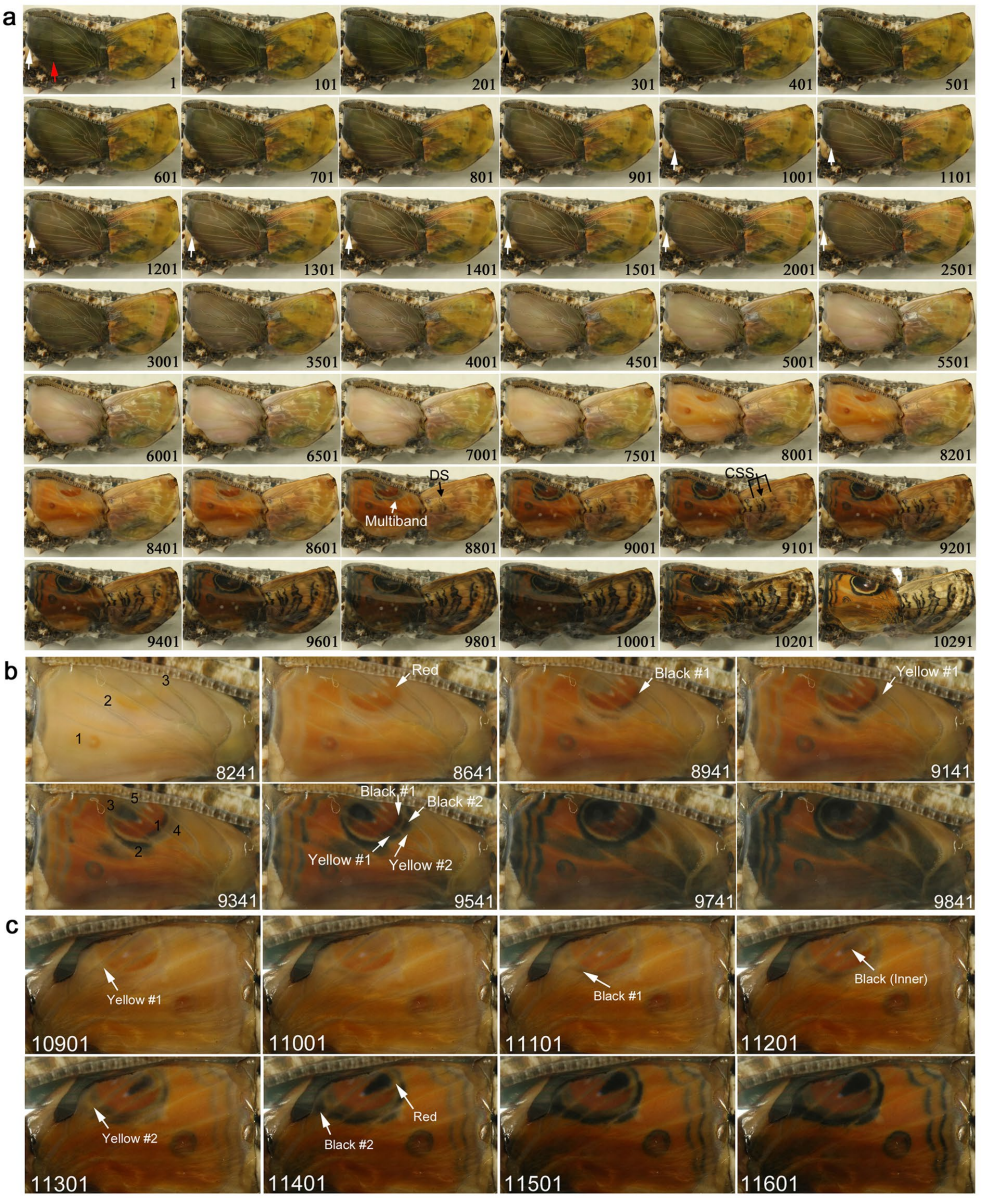
Developmental time course of the pupal forewing and hindwing of *J. almana*. The panel numbers indicate time points in min after the beginning of image recording within 1 h post-pupation. Different individuals are presented in a, b, and c. (**a**) Dynamics of a whole forewing and a hindwing. The red arrow in panel 1 indicates the possible front edge of the ventral epithelium. White arrows indicate possible boundary between the forewing proper and the peripheral region to be degraded. The proximal side of the major eyespot forms multiple bands (indicated as Multiband in the panel 8801) that are not seen in the final colour pattern. DS in the panel 8801 indicates the discal spot. CSS in the panel 9101 indicates the central symmetry system. See also Supplementary Video 1. (**b**) A hindwing. The numbers in the panel 8641 indicate the order of red emergence. The numbers in the panel 9341 indicate the order of black emergence. The black and yellow bands were numbered in the order of emergence from inside to outside. The Yellow #2 was overpainted entirely by the Black #2. See also Supplementary Video 2. (**c**) Another different hindwing with similar overpainting of multiple bands. A red area located at the distal portion of the major eyespot was also overpainted by the black area elongated from the central part of the major eyespot.

We first recorded the hindwing development of *J. almana* (*n* = 4). We succeeded in having kept both forewing and hindwing alive in one *J. almana* case (Fig. 1a; Supplementary Video S1). The four developmental stages (plus the pre-eclosion stage) were observed in *J. almana*, based on the hindwing colouration, although the exact transition time point from one stage to the next was difficult to specify because of gradual changes: the transparent stage (the first stage) (Fig. 1a, Panels 1–501), the semi-transparent stage (the second stage) (Panels 601–3001), the white stage (the third stage) (Panels 3501–7501), and the colouration stage (the fourth stage) (Panels 8001–10001). After the completion of development, there was the pre-eclosion stage (the fifth stage) (Panels 10201–10291).

The distal edge of the fore- and hindwings dynamically moved in the first and second stage, which may indicate a degradation of the peripheral portion by apoptosis^1,52–56^ and an expansion of the wing tissue proper through an increase of the number of cells (or through an increase of cellular volume with polyploidisation). A tissue underneath the dorsal epithelium of the hindwing, likely the ventral epithelium of the hindwing, moved towards the wing peripheral side, and expansion of the dorsal hindwing proper (corresponding to the adult dorsal hindwing) was also observed (Panels 1–2501). This expansion of the dorsal and ventral epithelia accompanied the slow low-frequency contractions in the first and second stages. The contractions gradually stopped at the end of the second stage, followed by the third white stage, which lasted relatively long. At this point, tracheae appeared to be loosened.

During the colouration stage, the proximal side of the major eyespot appeared to have multiple bands (Panel 8801). Forewing development appeared to lag behind hindwing development. In the ventral forewing, the discal spot and its associated bands of the central symmetry system emerged first (Panels 8801–9101), and the border and basal symmetry system appeared later.

The colouration sequence was further examined in different individuals in more detail (Fig. 1b; Supplementary Video S2). The red colouration emerged first in the posterior side and then expanded to the anterior side (Fig. 1b, Panel 8241). The black colouration then emerged, following the light-to-dark rule. In contrast, the sequence of black appearance was not simple. The basal black ring emerged first, from which the black region expanded to many directions (Panel 9341). After the red region of the major eyespot clearly emerged, the black line emerged immediately basal to the red region (Panel 8941). In the further basal area, the yellow line emerged (Panel 9141). The second black line then emerged to the further basal region, and then, another yellow line emerged (Panel 9541). These double yellow lines, which were not seen in the adult wings and thus may be called transient patterns, disappeared by the overpainting of the expanding black area (Panels 9741–9841). A similar sequence was observed in another individual (Fig. 1c). In addition to the anterior side of the major eyespot, the posterior side showed that the red area was also overpainted by the black area (Fig. 1c, Panels 11401–11601).

### Peripheral adjustment of the *J. almana* forewing

**Figure 2:**
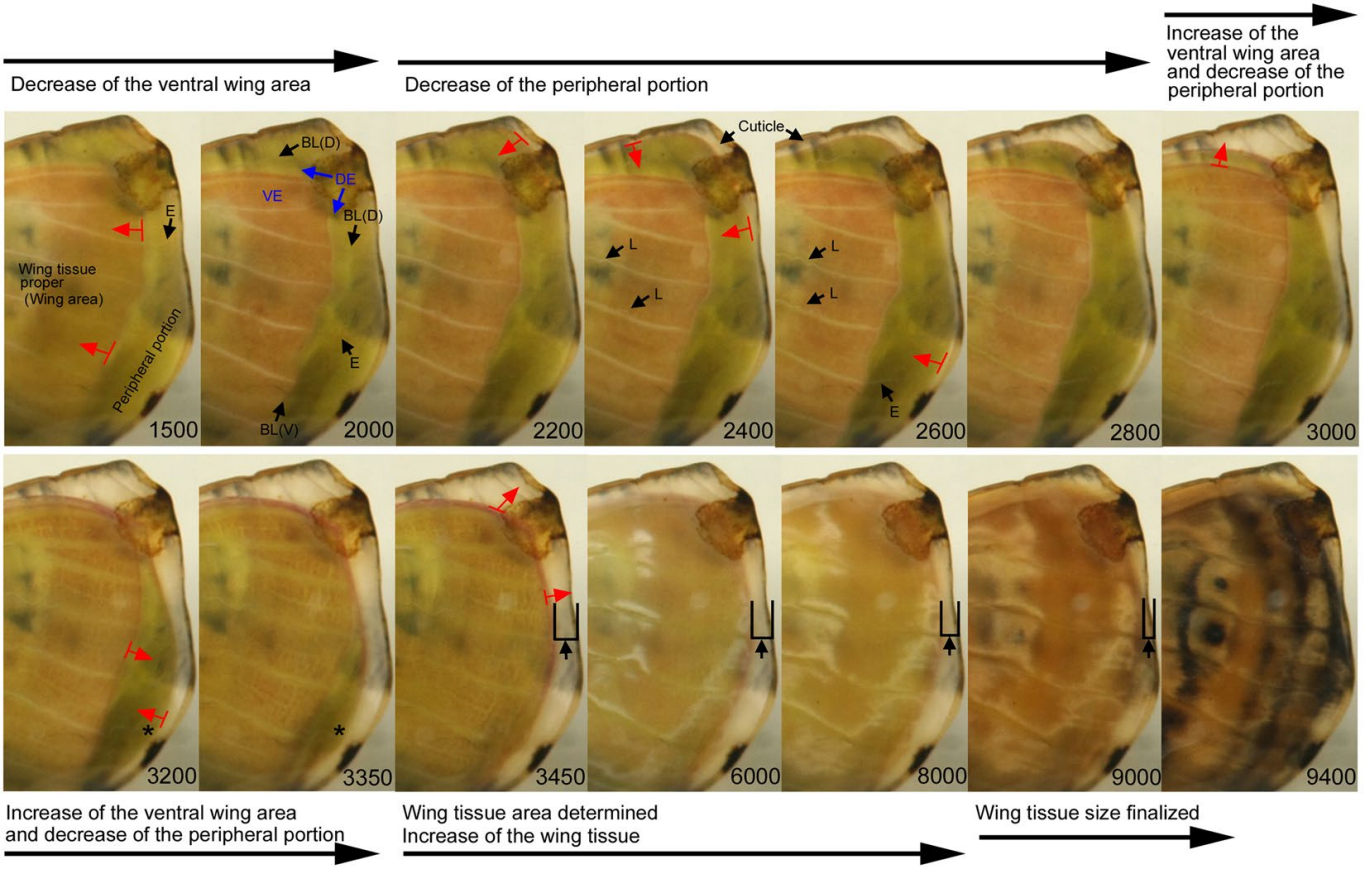
Peripheral adjustment of the pupal forewing of *J. almana*. This is the same individual as that in Fig. 1a. The panel numbers indicate time points in min after the beginning of image recording within 1 h post-pupation. See also Supplementary Video 2. The moving directions are shown in red arrows. Asterisks in the panels 3200 and 3350 indicate the peripheral portion to be degraded. Assuming that the pupal cuticle does not change in size, the gap distance between the edges of the pupal wing cuticle and the live pupal forewing were indicated by the vertical upward black arrows with U-type size indicators (Panels 3450–9000). The dorsal epithelium for the wing proper (DE), the ventral epithelium for the wing proper (VE), the border lacuna for the dorsal epithelium (BL(D)), the border lacuna for the ventral epithelium (BL(V)), extended trachea to the peripheral portion (E), and loosened trachea (L) are indicated.

As the movement of the hindwing epithelia detected thus far was complex, we focused on the movement of the forewing epithelia in detail. Fortunately, the ventral forewing proper (corresponding to the adult ventral forewing) was naturally seen in pink in this species, which allowed us to precisely record and interpret its movement (Fig. 2; Supplementary Video S1). At first, the ventral forewing proper diminished slightly in size, detected as an inward movement of the bordering lacuna (wing edge) for the ventral forewing proper (Panels 1500–2000). The dorsal forewing proper did not appear to change. Then, the peripheral portion (outside the forewing proper) clearly diminished in size, leaving the pupal cuticle behind (Panels 2200–2800). However, the ventral forewing proper then increased in size, while the peripheral portion further decreased. The dorsal and ventral epithelia (the wing area proper) then became completely overlapping (Panel 3450). After the elimination of the peripheral portion, the entire wing tissue expanded again until immediately before the pigment deposition process. This peripheral adjustment or similar dynamics was observed in all species examined in this study, although it was less clear than in the forewing of *J. almana*.

### Hindwing eyespot development in *J. orithya*

**Figure 3:**
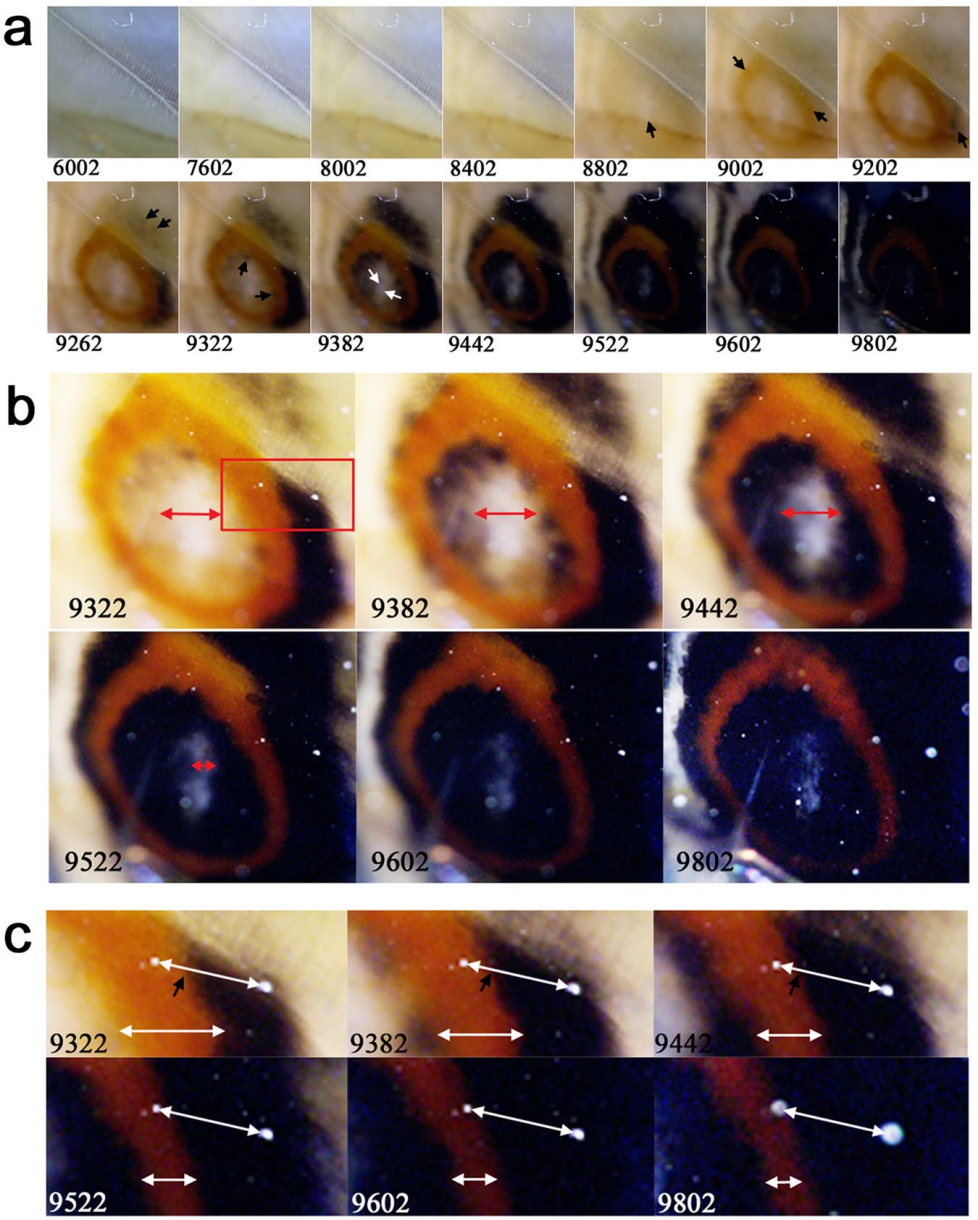
Developmental time course of the pupal hindwing eyespot of *J. orithya* in the CuA_1_ compartment. The panel numbers indicate time points in min after the beginning of image recording within 1 h post-pupation. (**a**) Colouration sequence of the eyespot development. A black arrow in the panel 8802 indicates the position of the eyespot focal white scales. Other black arrows indicate emergence of red or black areas. White arrows in the panel 9382 indicate the width of the focal white area, which diminishes in the following panels. (**b**) High magnification of the eyespot shown in a. The width of the focal white area is indicated by double-headed arrows, which diminish consecutively, showing an overpainting process. The boxed area is enlarged in c. (**c**) High magnification of the eyespot rings is shown in b. Upper double-headed arrows bridge two positional reference points (dusts on the cover film). The red-black boundary on the double-headed arrows is indicated by small black arrows. The positions of the boundary change in the next panels. Lower double-headed arrows indicate the width of the red ring, which diminishes in the next panels. These changes demonstrate the overpainting dynamics of colour pattern development.

Although some developmental images of *J. orithya* are already available in the literature^21,48^, we reanalysed images of the hindwing eyespot development of *J. orithya* that were obtained previously^48^ in more detail to examine the overpainting process (*n* = 3) (Fig. 3a). The focal white area, constructed by structural colouration, emerged as early as the red ring (Fig. 3a, Panel 8802). The black areas emerged as patchy islands in both sides of the red ring (Fig. 3a,b). The black areas soon expanded to form rings inside and outside the red ring (Fig. 3a, Panels 9202–9382; Fig. 3b,c). Because of the black expansion, the red ring was made narrower from both sides (Fig. 3a–c). The focal white area was also made narrower (Fig. 3a,b).

### Hindwing and forewing development of *Vanessa indica*

**Figure 4:**
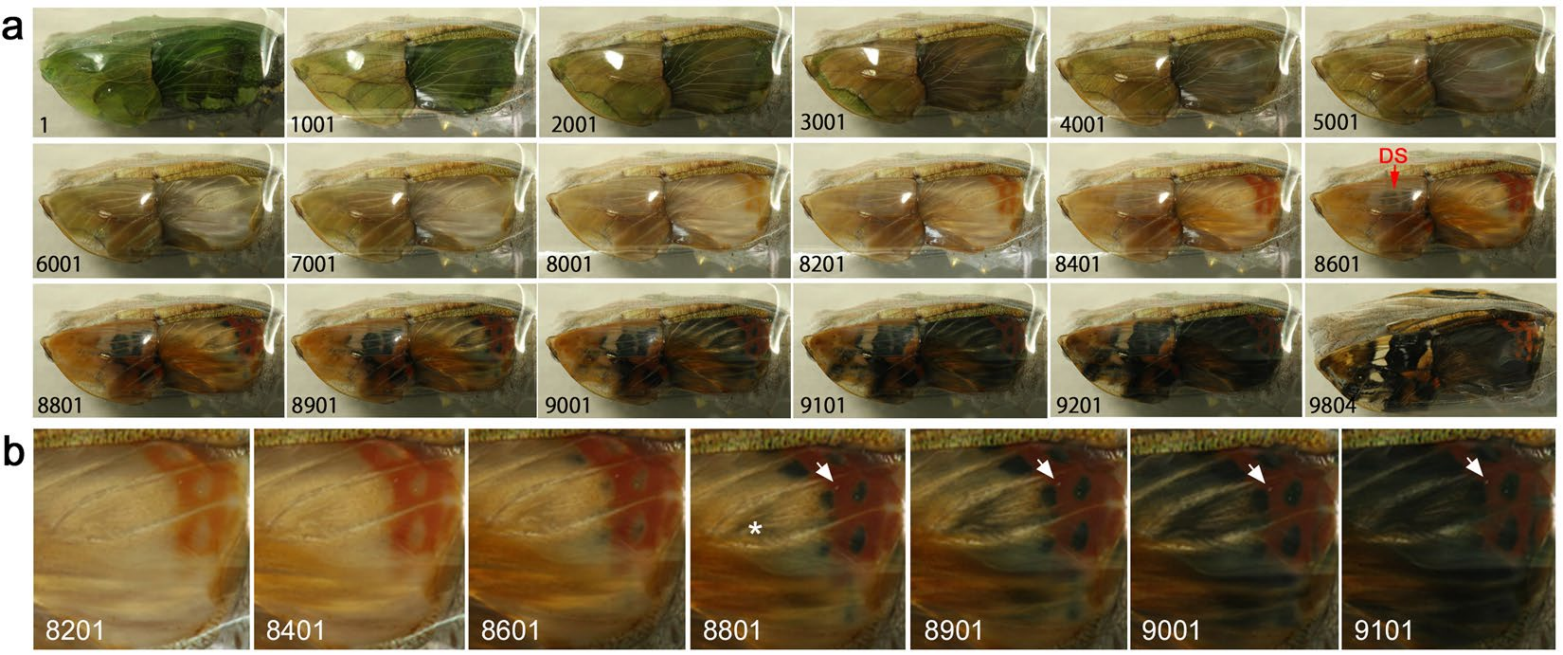
Developmental time course of the pupal forewing and hindwing of *V. indica*. The panel numbers indicate time points in min after the beginning of image recording within 1 h post-pupation. (**a**) Time course of the forewing and hindwing development. DS indicates the discal spot emerged on the ventral forewing. See also Supplementary Video 3. (**b**) Higher magnification of a. Red emerged first, followed by black in the border symmetry system and its surroundings in the dorsal hindwing. Arrows indicate the positional reference point (a dust on the cover film), indicating the invasion of the black area into the red area. An asterisk indicates one of the black emerging points that may not belong to the border symmetry system.

An additional species of Nymphalinae, *Vanessa indica*, was examined (*n* = 2). In one case, both forewing and hindwing were alive (Fig. 4a; Supplementary Video S3). Developmental changes were largely similar to those of *Junonia* butterflies above. For example, the peripheral adjustment was observed in both fore- and hindwings but more clearly in the forewing. The red colouration emerged first in the hindwing (Fig. 4a, Panels 8001–8401), and then, the black spots corresponding to the eyespots and the parafocal elements appeared (Panels 8601–9201), following the light-to-dark rule. These black spots expanded and invaded the red area, exhibiting the overpainting (Fig. 4b). The discal spot and its associated bands of the central symmetry system emerged first in the ventral forewing (Fig. 4a, Panel 8601), and other elements appeared later. These results largely confirmed the previous findings in *J. almana* and *J. orithya*.

### Forewing development of *Argyreus hyperbius* and *Athyma selenophora*

**Figure 5:**
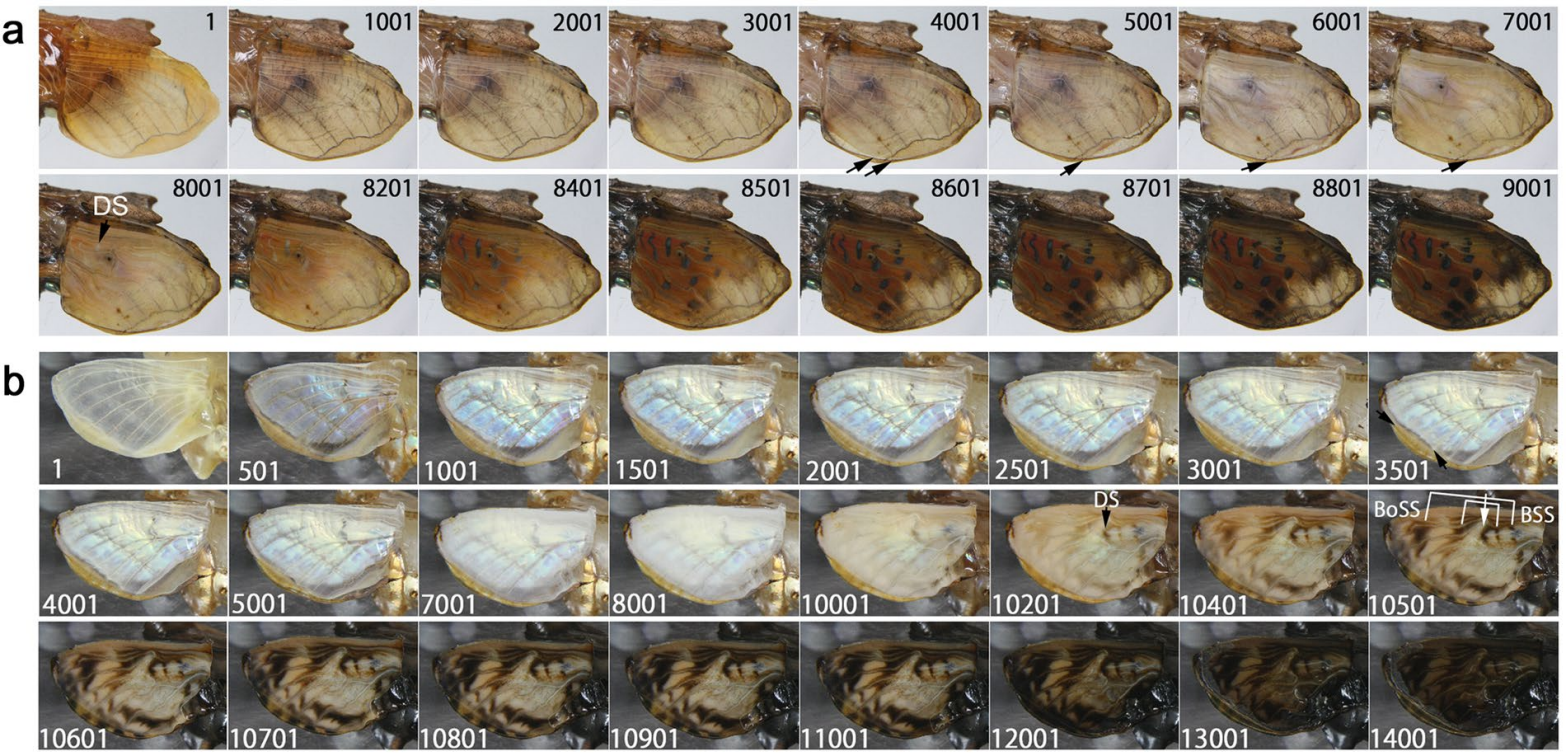
Developmental time course of the pupal forewing of *Argyreus hyperbius* and *Athyma selenophora*. The panel numbers indicate time points in min after the beginning of image recording within 1 h post-pupation. (**a**) *Argyreus hyperbius*. Arrows in the panel 4001 indicate the edges of the dorsal and ventral epithelia. Arrows in the following panels indicate the forewing edge after the alignment of the dorsal and ventral epithelia. However, the edge was not aligned with the wing edge specified by the cuticle lines. DS in the panel 8001 indicates the discal spot. See also Supplementary Video 4. (**b**) *Athyma selenophora*. The cuticle of this pupae is metallic gold, and thus, some reflections cannot be avoided. In the panel 10201, the DS is indicated. In the panel 10501, the border symmetry system (BoSS) and the basal symmetry system (BSS) are shown. See also Supplementary Video 5.

Two butterfly species that belong to different subfamilies, Heliconiinae and Limenitidinae, were here examined. In these species, the hindwing was difficult to save for unknown reasons, but the forewing was often saved to complete wing development, where several black elements exist. In *Argyreus hyperbius* (*n* = 1), the peripheral adjustment was clearly observed (Fig. 5a; Panels 4001–7001; Supplementary Video S4). The reddish brown background expanded from the basal to peripheral areas, slightly before the emergence of the black colour (except the discal spot) (Fig. 5a, Panel 8001), following the light-to-dark rule. The discal spot emerged first among the black spots, and other black spots emerged around the discal spot (Panels 8001–9001). In *Athyma selenophora* (*n* = 1), the peripheral adjustment was also observed (Fig. 5b; Panels 3501–8001; Supplementary Video S5). Again, the discal spot emerged first (Panels 10001–10201), and then, its associated central symmetry system emerged (Panels 10001–10201), followed by the basal symmetry system and the border symmetry system (Panels 10201–12001). It appeared that these two systems formed a circular and continuous ring around the discal spot.

### Forewing development of *Danaus chrysippus* and *Danaus genutia*

**Figure 6:**
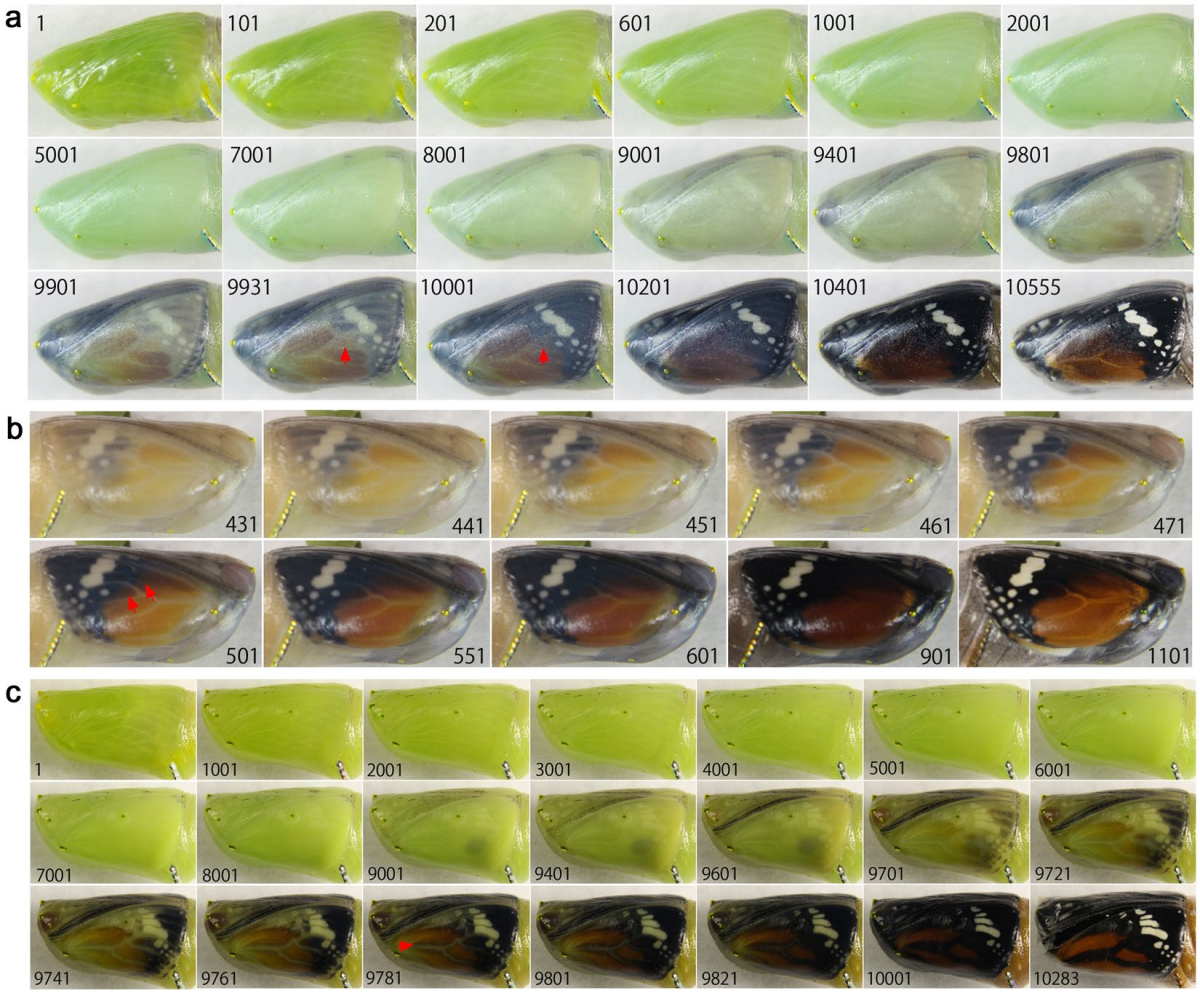
Developmental time course of the pupal forewing of two closely related species, *D. chrysippus* and *D. genutia*. The panel numbers indicate time points in min after the beginning of image recording within 1 h post-pupation except b. (**a)** *D. chrysippus*. Red arrows in panels 9931 and 10001 indicate the areas that are soon invaded by the black expression. See also Supplementary Video 6. (**b**) A different individual of *D. chrysippus*. Red arrows in the panels 461 and 471 indicate the areas that are soon invaded by the black expression. See also Supplementary Video 7. (**c**) *D. genutia*. An arrow in the panel 9781 indicates an emerging vein-dependent black area that later expands to overwrite the surrounding red area. The vague black circles seen in panel 9001 and later in the wing area are not wing pigments but were often observed in this species. See also Supplementary Video 8.

Two closely related species of Danainae, *D. chrysippus* (Fig. 6a,b; Supplementary Videos S6, S7) and *D. genutia* (Fig. 6c; Supplementary Video S8) were examined. These species have similar colour patterns, but the latter had distinct black wing veins. Pupae of these species were largely transparent, so that the dorsal forewing development was observed without surgical operation. In *D. chrysippus* (*n* = 4), the white band emerged first (Panel 9001), and the red (reddish brown) and black pigments emerged simultaneously (Fig. 6a, Panel 9801), which was an exception of the light-to-dark rule for ontogenic colouration. The black colour for the wing tip area appeared strongly and “spread” over the posterior and basal areas (Fig. 6a, Panels 9801–10001; Fig. 6b, Panels 431–551). The red area also spread from the distal to the proximal areas. Importantly, some areas that were coloured in red first were painted in black later (Fig. 6a, Panels 9931–10001; Fig. 6b, Panels 471–501). This overpainting occurred at the boundary between the black and red colours. In *D. genutia* (*n* = 2), the white band emerged first (Fig. 6c, Panel 9601), and the red and black pigments emerged simultaneously (Panel 9701). The overpainting at the red-black boundary was less clear than that in *D. chrysippus*. However, wing veins were blackened relatively later on both sides of the wing vein (Panel 9781), and this black area expanded and invaded the surrounding red areas.

### Forewing and hindwing development of *Idea leuconoe* and *Ideopsis similis*

**Figure 7:**
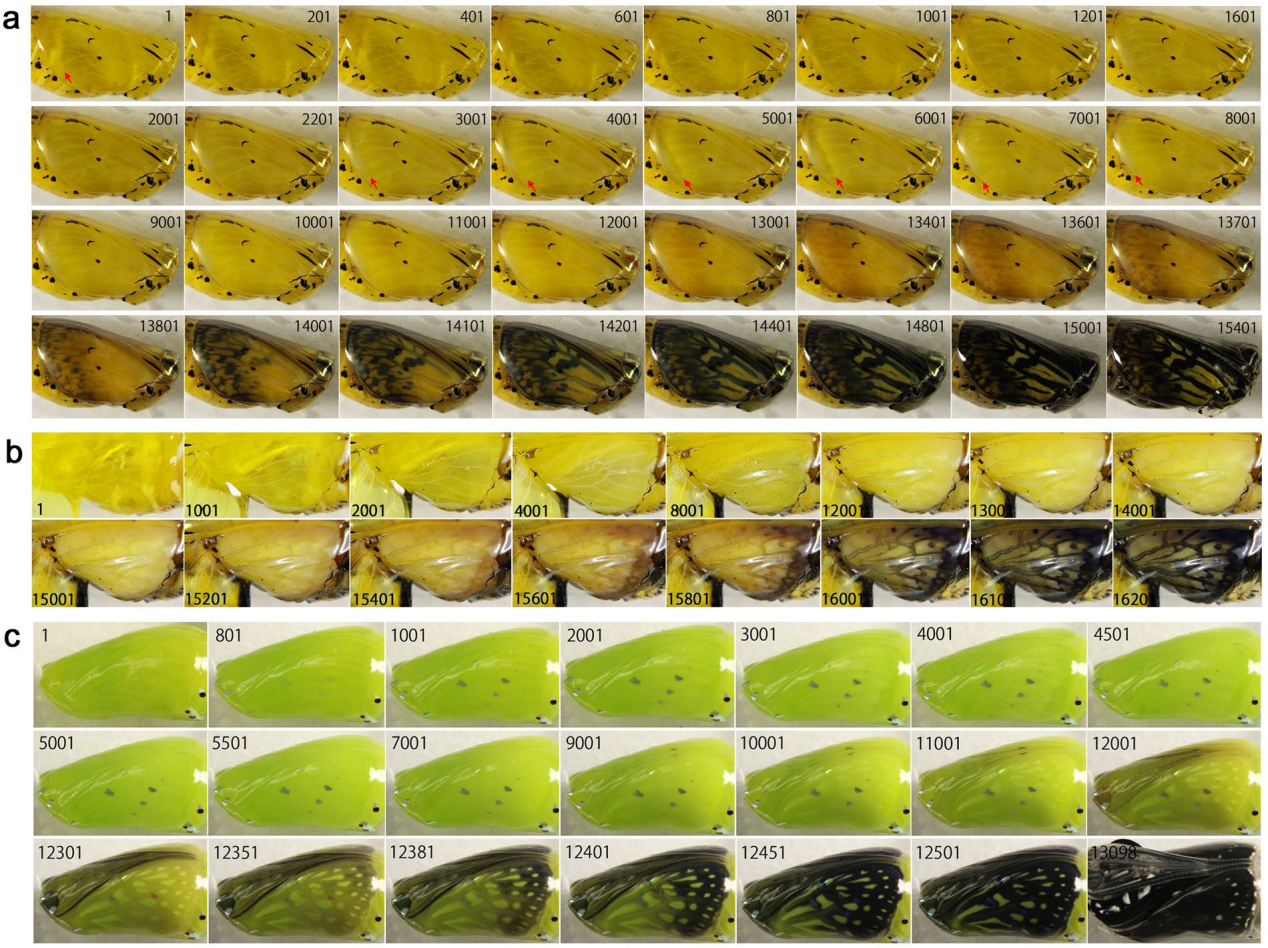
Developmental time course of the pupal forewing and hindwing of *Idea leuconoe* and *Ideopsis similis*. The panel numbers indicate time points in min after the beginning of image recording within 1 h post-pupation except a and c. (**a**) Forewing of *Idea leuconoe*. Arrows indicate edges of the ventral epithelium. See also Supplementary Video 9. (**b**) Hindwing of *Idea leuconoe*. See also Supplementary Video 10. (**c**) Forewing of *Ideopsis similis*. See also Supplementary Video 11.

Two additional Danainae species were examined. Pupae of *Idea leuconoe* are usually metallic gold, but we fortunately obtained transparent pupae of this species, so that the real-time developmental observations were possible without the forewing-lift procedure (*n* = 1). In this species, the peripheral adjustment was observed in the forewing (Fig. 7a; Supplementary Video S9). The black spots appeared first at the distal portion of the forewing and spread over to the proximal portion. The wing veins were blackened after the emergence of black spots nearby. The discal spot did not emerge first in this species. The forewing-lift procedure was also performed, so that the dorsal hindwing development was observed (*n* = 1). The results were essentially the same; the distal portion was first coloured, and the colouration area spread over to the proximal portion (Fig. 7b; Supplementary Video S10). Similarly, in *Ideopsis similis* (*n* = 3), the distal portion was first blackened, and the black area spread over to the proximal portion (Fig. 7c; Supplementary Video S11), although the black pigment emerged simultaneously throughout the wing.

### Forewing and hindwing development of *Zizeeria maha* and *Lycaena phlaeas*

In addition to the 9 nymphalid species examined thus far, two species from the family Lycaenidae were here examined. Both forewing and hindwing development was observed in the forewing-lift configuration. Additionally, colour development of the dorsal forewing was observed without surgical operation through semi-transparent pupal cuticle.

**Figure 8:**
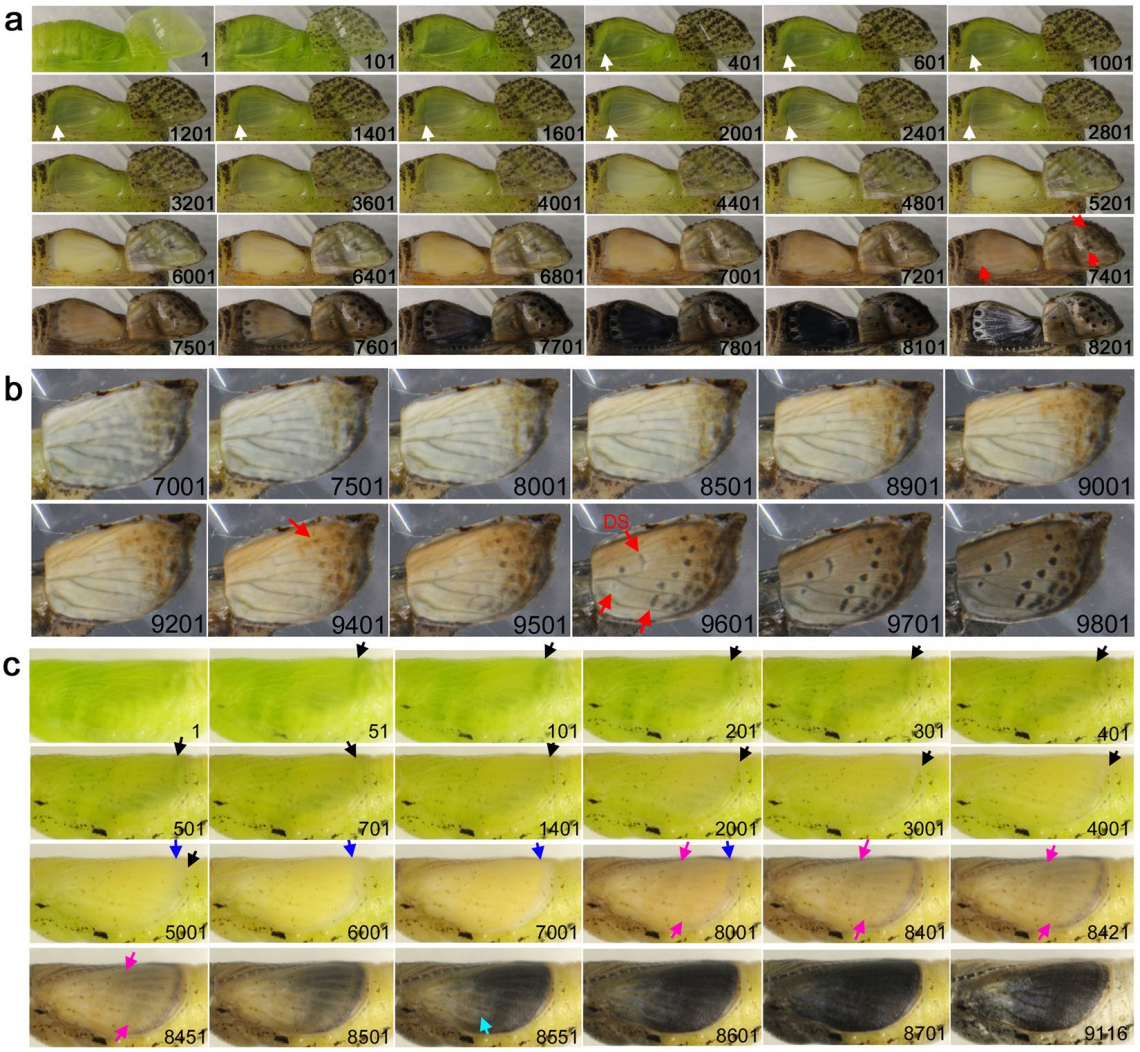
Developmental time course of the pupal forewing and hindwing of *Z. maha*. The panel numbers indicate time points in min after the beginning of image recording within 1 h post-pupation. (**a**) Ventral forewing and dorsal hindwing development of *Z. maha argia* (from Osaka). White arrows indicate moving edge of the dorsal hindwing epithelium. Red arrows indicate emerging black spots. See also Supplementary Video 12. (**b**) Ventral forewing colouration of *Z. maha argia* (from Osaka). Red arrows indicate emerging black spots. DS, discal spot. See also Supplementary Video 13. (**c**) Dorsal forewing development of *Z. maha okinawana* (from Okinawa) without operation. Black arrows indicate moving edge of the ventral epithelium. Blue arrows indicate newly defined forewing edge. Pink arrows indicate emerging black bands of the central symmetry system. Light blue arrow indicate structural colour. See also Supplementary Video 14.

In *Z. maha* (*n* = 2), the four sequential developmental stages were observed (Fig. 8a,b; Supplementary Videos 12, 13), similar to the nymphalid butterflies. In the first and second stages, the peripheral dynamics was observed both in the forewing-lift configuration (*n* = 2) (Fig. 8a,b) and without the operation (*n* = 1) (Fig. 8c). In the dorsal hindwing, the black spots at the wing margin first emerged (Fig. 8a, Panel 7401), and the black region expanded later. In the ventral forewing, the black spots at the wing margin also emerged first (Fig. 8a, Panel 7401; Fig. 8b, Panel 9401), and the proximal black spots including the discal spot emerged later (Fig. 8b, Panel 9601). In contrast, in the dorsal forewing, the black area emerged first as two anteroposterior parallel arrays (Fig. 8c, Panels 8001–8451; Supplementary Video 14) and expanded in proximodistal direction. The structural bluish colour then emerged (Panels 8501–8551).

**Figure 9:**
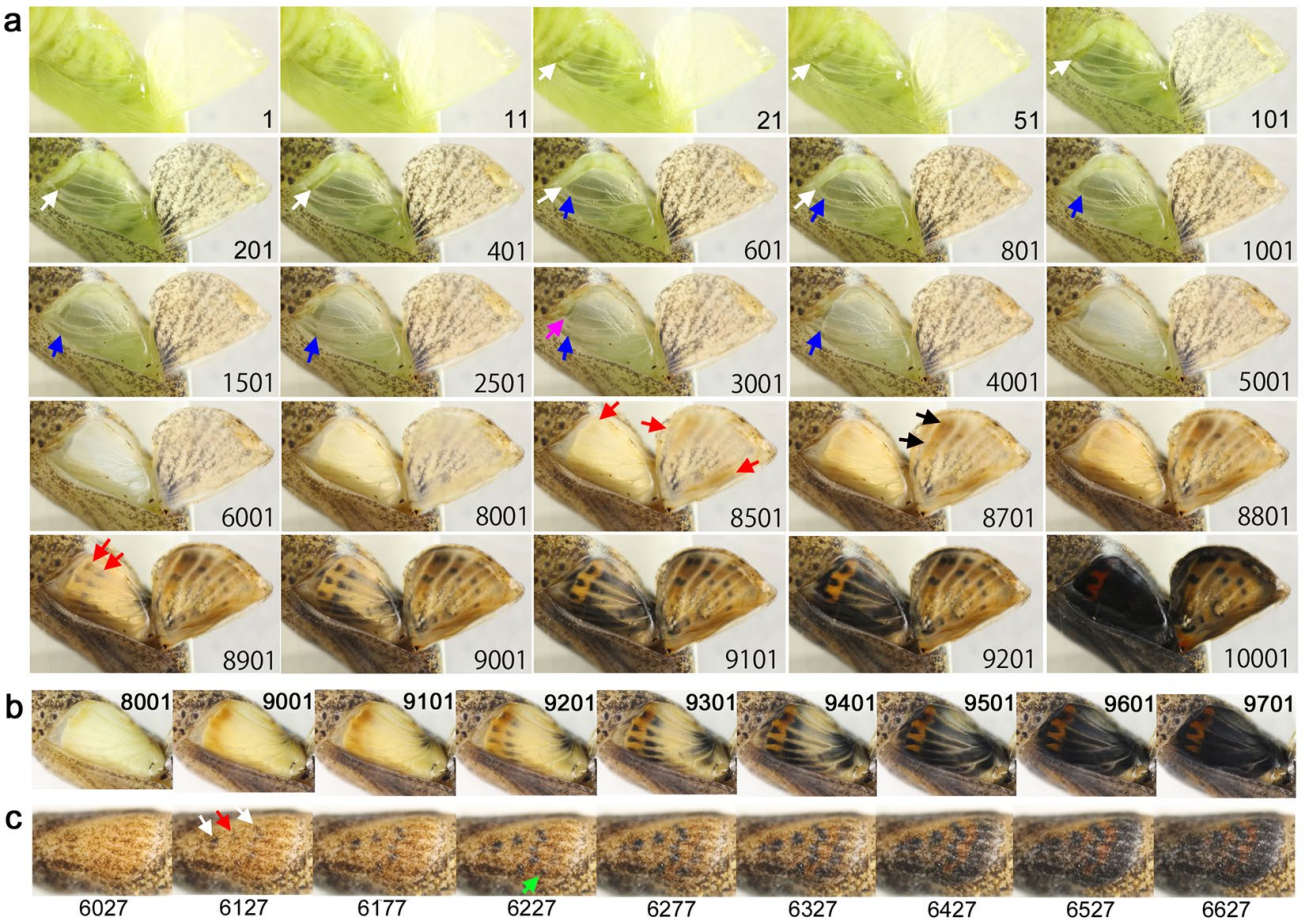
Developmental time course of the pupal forewing and hindwing of *L. phlaeas*. The panel numbers indicate time points in min after the beginning of image recording within 1 h post-pupation except c. (**a**) Ventral forewing and dorsal hindwing development. White arrows indicate moving edge of the ventral hindwing epithelium. Blue arrows indicate moving edge of the dorsal hindwing epithelium. Pink arrow indicates retracted edge of the ventral hindwing epithelium in Panel 3001, after which the edges of the dorsal and ventral epithelia appeared to be aligned. Red and black arrows indicate emerging red and black areas, respectively. See also Supplementary Video 15. (**b**) Dorsal hindwing colouration. See also Supplementary Video 16. (**c**) Dorsal forewing development without operation. White and red arrows in Panel 6127 indicate emerging spots that constitute the central symmetry system. The red arrow indicates the discal spot. A green arrow in Panel 6227 indicates emerging marginal black area. The image recording was started approximately 39 h post-pupation, and this time point was the starting point of the panel numbers of this figure and corresponding Supplementary Video 17.

In the hindwing of *L. phlaeas* (*n* = 2), the four sequential stages were observed, and the peripheral adjustment was observed in the first and second stages (Fig. 9a; Supplementary Videos 15, 16), as in the case of *Z. maha* above. In the second stage, the slow low-frequency contractions were also observed. The red colour was deposited first at the marginal area (Fig. 9a, Panels 8501–8801; Fig. 9b, Panels 8001–9001), and the black spot emerged within the red area (i.e., the direct overpainting) and at the proximal side of the red area. In the ventral forewing (*n* = 1), red colouration emerged first, as in the dorsal hindwing, and the red and black areas expanded from the tornus to the anterior and basal sides (Fig. 9a, Panels 8501 and subsequent ones). In the dorsal forewing (*n* = 1), red colouration appeared to be deposited first (Fig. 9c, Panel 6027; Supplementary Video 17). Soon after that, the arrays of the black spots that constitute the central symmetry system including the discal spot emerged (Fig. 9c, Panels 6127–6177), and the marginal black area emerged later (Fig. 9c, Panels 6227–6327). This colouration sequence is different from those of the ventral forewing and the dorsal hindwing of this species, but it is similar to that of the dorsal forewing of *Z. maha*.

### Forewing and hindwing development of *Pieris rapae*

**Figure 10:**
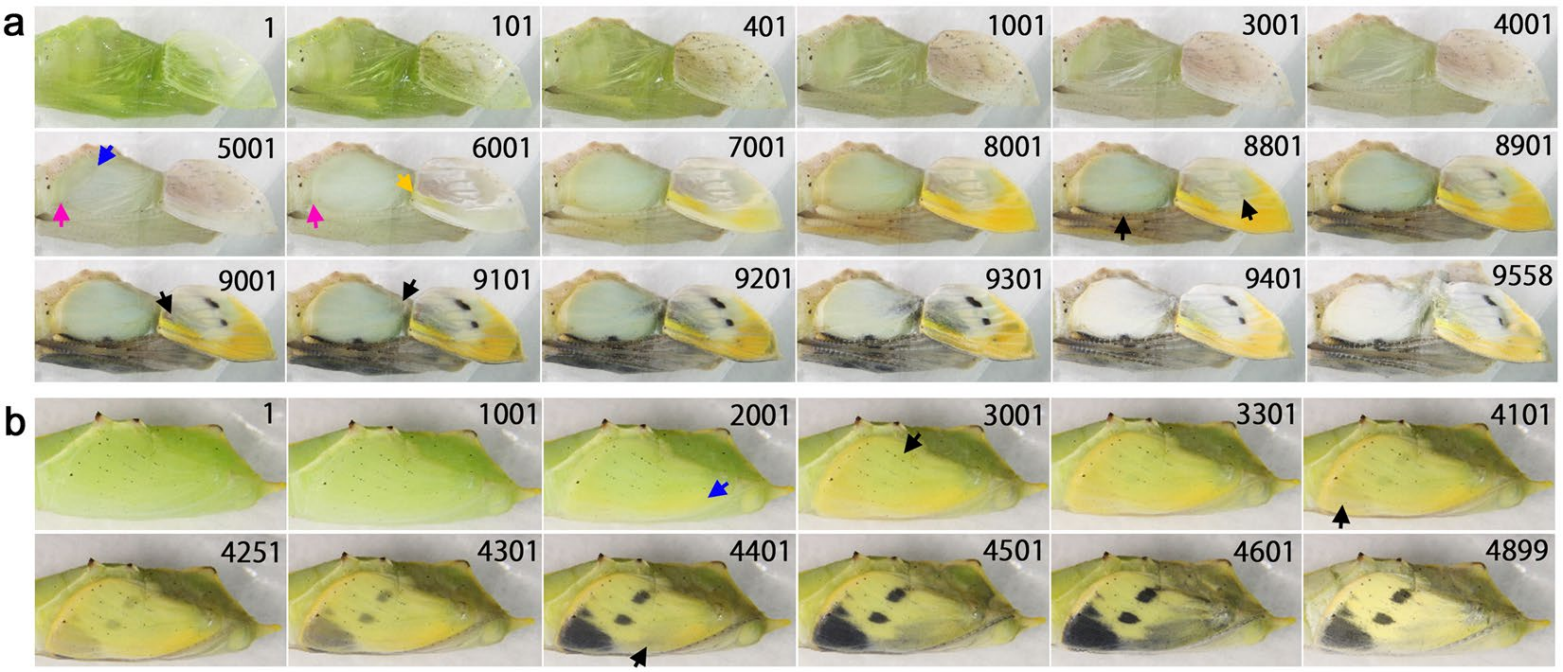
Developmental time course of the pupal forewing and hindwing of *P. rapae*. The panel numbers indicate time points in min after the beginning of image recording within 1 h post-pupation. (**a**) Ventral forewing and dorsal hindwing development. Pink arrows in Panels 5001 and 6001 indicate moving edge of the ventral hindwing epithelium. A blue arrow in Panel 5001 indicates moving edge of the dorsal hindwing epithelium. A yellow arrow indicates emerging yellow area. Black arrows indicate emerging black area. See also Supplementary Video 18. (**b**) Dorsal forewing development without operation. A blue arrow indicates emerging yellow area. Black arrows indicate emerging black area. The central black spots in the middle of the forewing did not seem to overpaint any yellow area. See also Supplementary Video 19.

Lastly, we examined one species from the family Pieridae, *Pieris rapae*, in the forewing-lift configuration (*n* = 1) and without the operation (*n* = 1). The peripheral adjustment, the slow low-frequency contractions, and the four sequential stages that were observed above were also observed in this species (Fig. 10a; Supplementary Video 18). In the ventral forewing, the marginal yellow area emerged first (Fig. 10a, Panel 6001), and then the black spots followed (Panel 8801), following the light-to-dark rule. Similar sequence was observed in the dorsal forewing (Fig. 10b; Supplementary Video 20). Interestingly, the marginal yellow area that emerged first (Fig. 10b, Panel 2001) mostly changed (i.e., directly overpainted) to the black area that emerged at the apex (Panel 4101) and along the anterior margin (Panel 4401) in the dorsal forewing. However, there was no such yellow area corresponding to the central black spots before their emergence in the middle of the dorsal forewing.

## Discussion

In this study, 9 nymphalid butterfly species from Nymphalinae, Heliconiinae, Limenitidinae, and Danainae were examined for their wing development in pupae. We were unable to include species from Satyrinae, one of the major subfamilies of Nymphalidae, in the present study for technical reasons (i.e., they were not very resistant to the operation). Additionally, 2 lycaenid and 1 pierid butterfly species were examined. To our knowledge, real-time *in vivo* whole wing image recordings that were presented in this study are the most comprehensive visualisation results of butterfly wing development at the whole wing level known to date. We believe that these records are crucial to understanding the possible mechanisms of wing development and colour pattern determination in butterflies.

We were able to observe the phenomena that have been reported in previous studies^1,21,27,29,48^ and that have never been reported before. The findings common in the 12 butterflies examined are the four stages of the wing development (although this was not clear in Danainae samples without the forewing lift), dynamic peripheral wing size adjustment including decrease and increase of the wing area proper, slow low-frequency contractions of the entire wing tissues, and overpainting. These dynamic changes have profound implications in understanding wing development as well as colour pattern determination mechanisms at the tissue level. The patchy island formation and subsequent expansion for colouration detected in *J. orithya* should be examined in other systems in the future.

**Figure 11:**
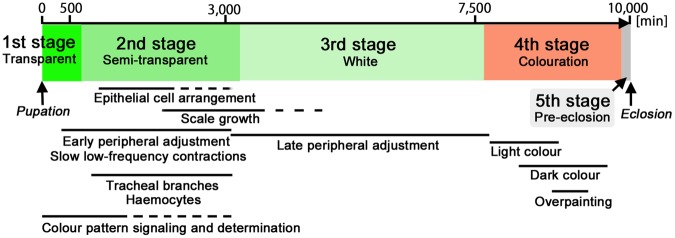
Time table of pupal wing development. This time table is just a rough summary of the findings of the present study, assuming that the entire time span from pupation to eclosion is approximately 10,000 min (7 days). Some important events are also indicated based on the previous real-time imaging study using *J. orithya*^48^.

Four stages and their associated events were summarised in Fig. 11. These stages were commonly observed in all species studied here at least in the hindwing, although relative stage durations may vary. Interestingly, all species spent roughly 10,000 min (7 days) before eclosion. The period of intense colour pattern signaling, roughly within 6–12 h (360–720 min) post-pupation, may coincide roughly with the first stage, but the period of the entire colour pattern signaling and determination may correspond to the first and second stages^1,5,23^. It is somewhat surprising to recognise that the colour pattern signaling and determination occur in these very dynamic stages. The second stage has several marked events, and the cease of the contractions roughly marks the end of the stage. Also at the end of the second stage, the dorsal and ventral epithelia for the wing area proper became completely overlapping. The third stage is relatively static, but the white level gradually becomes intense. Colouration occurs in the fourth stage, following the light-to-dark rule, and resulting in the overpainting. A similar time table based on classical histological studies can be found in Figure 1.14 of Nijhout (1991)^1^. Important differences from the classical studies are recognition and discovery of dynamics; dynamic changes of the peripheral adjustment, tissue contractions, secondary tracheal branches, haemocytes, epithelial cell arrangement, scale growth, and overpainting.

Colouration sequence (ontogeny of the pigment deposition) has been said to be dependent on biochemical synthesis pathways because light (mostly ommochromes) and dark (mostly melanins) pigments are chemically different^1,29^. In this study, light colours emerged before dark colours in *J. almana*, *J. orithya*, *V. indica*, *Argyreus hyperbius*, *L. phlaeas*, and *P. rapae*, as expected based on the previous studies^1,29^. Thus, the present study confirmed that the light-to-dark rule is applicable to various species belonging to Nymphalidae (Nymphalinae and Heliconiinae), Lycaenidae (Lycaeninae), and Pieridae (Pierinae). However, this rule did not seem to be applicable strictly to *D. chrysippus* and *D. genutia*, two closely related species of Danainae, in which red and black emerged simultaneously at least at the resolution of this study (i.e., the highest time resolution was one minute).

In species where the light-to-dark rule is applicable, there is no reason to believe that the emerging order among elements and sub-elements of different colours reflects the order of colour fate determination among elements because the order of emergence may simply follow the order of completion of pigment synthesis. However, the emerging order among the elements and sub-elements of the “same colour” may indicate the order of fate determination. Indeed, in the forewings of the 4 nymphalid species *J. almana*, *V. indica*, *Argyreus hyperbius*, and *Athyma selenophora*, the elements that have the same colour did not emerge simultaneously but emerged at different time points. It appears that the discal spot (the core element of the central symmetry system) emerged first and the basal and border symmetry systems emerged later. This emerging order may reflect the functional importance of the discal spot organiser in positioning the elements of the three major symmetry systems wing-wide. In contrast, in all of the 4 Danainae species that were examined, the colouration was first executed in the white band and at the distal portion, and the coloured area gradually expanded towards the proximal portion. This colouration sequence showed no priority for the discal spot (although the discal spot is not well defined in Danainae in the first place) and is different from that of other nymphalid butterflies that were examined in this study. In Lycaenidae, the emerging order was different between the dorsal and ventral sides; the central symmetry system emerged relatively early in the dorsal forewing, but in the ventral forewing and dorsal hindwing, the marginal spots emerged earlier than the central symmetry system.

The discovery of the complex dynamics of the peripheral portions of the wings, here called the peripheral adjustment, may shed light on the mechanisms of wing shape formation and size determination. At the early stage, the peripheral portion of the wing tissue diminished in size to form the adult wing shape. This initial size reduction may be executed via apoptosis of the peripheral portion of the tissue^1,52–56^. It has been thought that the bordering lacuna defines the wing proper inside, and the peripheral portion outside the bordering lacuna degenerates. This process is often dubbed as a cookie-cutter-like mechanism^53^. However, this peripheral size reduction appeared to accompany loosening of tracheae in the wing proper, suggesting that the size reduction may also be caused by shrinkage of the entire tissue.

Surprisingly, the ventral wing proper shrank and then expanded even during the peripheral reduction. Because the dorsal epithelium secretes cuticle and tightly binds to it before apolysis (detachment of the dorsal epithelium from the pupal cuticle), the dorsal epithelium may not be able to move, as flexible as the ventral epithelium is at the early pupal stages. Thus, the shrinkage of the peripheral potion (Fig. 2, Panel 2200) may thus indicate apolysis as well as apoptosis, and it is likely that the ventral epithelium is independent of the dorsal epithelium in terms of size changes. The expansion of the wing tissue proper is likely in concert with the slow low-frequency contractions^48^ to finalise the tissue size by adjusting the number of scale rows (i.e., the number of scale cells) and scale cell size^57^.

Because the wing tissue is believed to be a flat sac-like structure with a continuum of the dorsal and ventral sides, this independent movement of the ventral epithelium is unexpected. It is possible that in butterflies, the continuum may be lost at the dorsoventral boundary (the peripheral portion) to allow each epithelium to behave independently at the early stage. Bordering lacuna may play a role in that process. Then, towards the final stage of the wing development, after the elimination of the peripheral portion and the complete alignment of the ventral and dorsal epithelia, the wing size expanded again. The tissue expansion at this stage may be involved in the enlargement of the entire wing. This final tissue expansion continued until immediately before the pigment deposition.

These dynamic movements were clearly observed in the forewings of *J. almana*, but both fore- and hindwings in all other butterflies that were examined in this study showed similar movements. These dynamic movements, including the contractions of the whole wing tissue, may be assisted by the wing hearts located in the thorax through haemolymph flow into the space between the dorsal and ventral epithelia^58^, but our observations here could not clarify a relationship between haemolymph flow and the wing movement. The wing clearing of haemocytes that were reported in *Drosophila*^59^ were not observed here. Higher resolution images in time and space may help clarify this issue.

Venation patterns of the dorsal and ventral epithelia of the wing coincide in adults. However, the venation patterns are first determined at the larval stage, and they are expressed later in the pupal stage^60,61^. The dorsal and ventral epithelial sheets interact with each other at the pupal stage when the basal laminae located between the two epithelia temporally degenerate^60,61^. This interaction has been thought to contribute to the matching of the dorsal and ventral venation systems^60,61^. The movements of the dorsal and ventral epithelia recorded in this study may contribute to this process.

**Figure 12:**
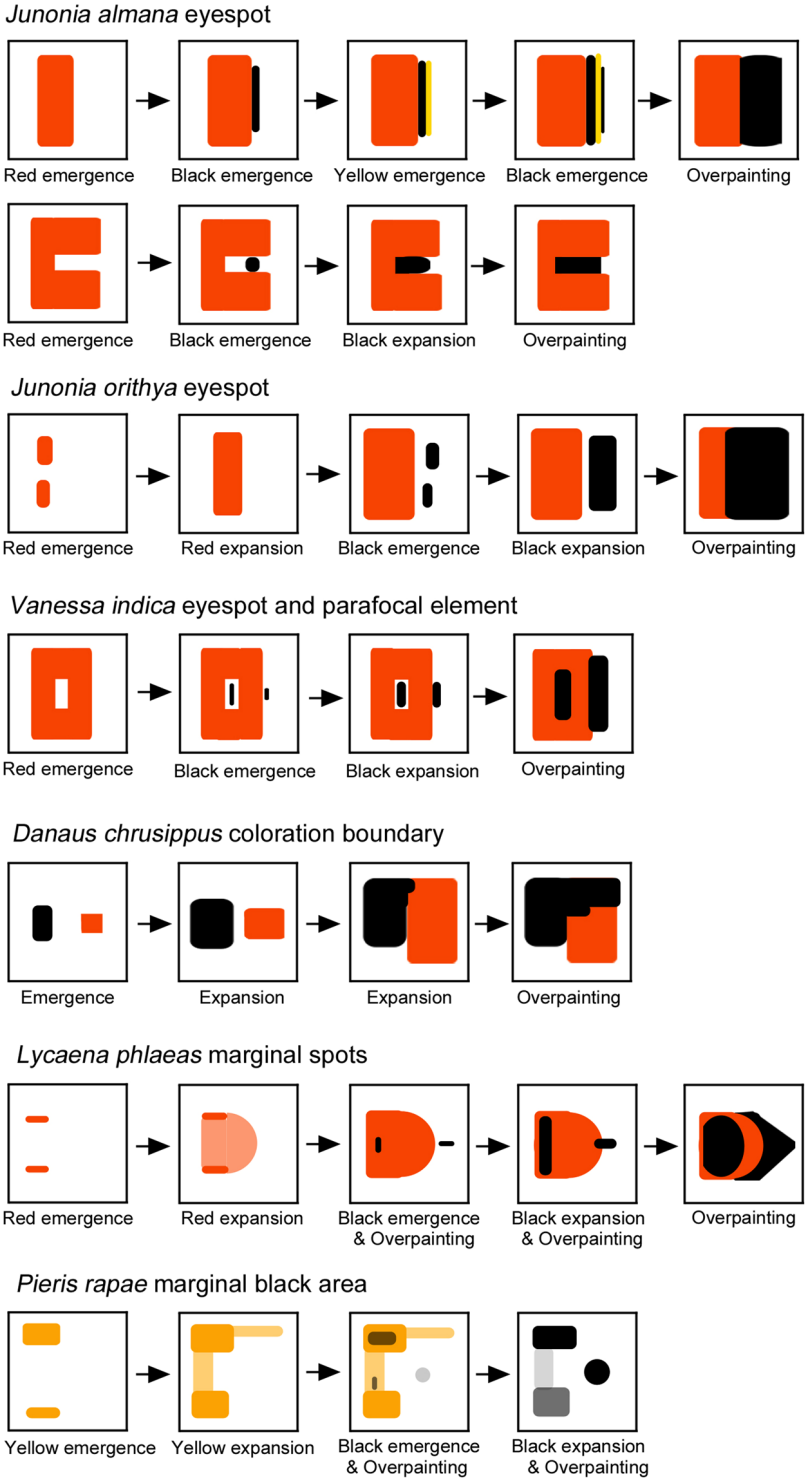
Schematic diagram of the overpainting. In *J. orithya*, red areas emerge as patchy islands, but they merge into a single red area as they expand. At the same time, black areas emerge as patchy islands. They also merge into a single black area as they expand. As the black area further expands, the red area is overpainted by the black area. In this species, the focal white area is also overpainted by the surrounding black area (not shown in this diagram). In *D. genutia*, an expansion of the vein-dependent black area also involves the overpainting (not shown in this diagram). In *L. phlaeas*, black area emerges in the middle of the red area. In *P. rapae*, most yellow areas change to black areas. The central black spots emerge without any previous yellow colouration.

Although the overpainting was briefly pointed out in a previous study^48^, the present study is the first systematic effort to research and present this phenomenon. Overpainting was clearly observed in 7 species examined in this study, and its time course was summarised in Fig. 12. In 5 nymphalid cases, the red or yellow colour was overpainted with black colour that expanded from the nearest black region. The red or yellow region is clearly established first, despite its transient existence, before the black invasion. In 2 other cases (i.e., lycaenid and pierid cases), black colour emerged at and expanded from the middle of the red or yellow colour area. The overpainted scales likely have two kinds of pigments (e.g., red and black pigments), but light (e.g., red) pigment is undetectable because of coexisting dark (e.g., black) pigment. This may violate the one-scale one-colour rule^1,5^, but this is not surprising, because this “rule” has not been rigorously tested before. Interestingly, we showed that in *J. orithya*, the white structural colour at the centre of an eyespot is also invaded by the black region. The white area is shown to be independent of other sub-elements, at least in *Calisto* butterflies^28^, but even in those butterflies, the intermediate scales that have both structural and pigment colours are detected, suggesting the coexistence of two different morphogenic signals in a scale cell to express two mixed colours^28^.

We believe that epithelial cells that had the overpainted scales had the positional information for dual colours, meaning that a mathematical model for colour pattern formation should allow this double coding per cell. A strict threshold model such as the classical gradient models for positional information, in which multiple thresholds to a single signal gradient determine the final colouration^22^, should be modified or abandoned accordingly. The induction model, which posits the lateral induction and inhibition as a basic mechanism of colour pattern formation^5,23,24^, may be more flexible because the signals for red (light colour) and black (dark colour) are different in entity and thus can be present simultaneously in a single cell. Indeed, some colouration sequences that were shown in the present study (especially that of the basal side of the major eyespot in *J. almana*) are reminiscent of a serial induction process that is observed in fish eyespots^62^.

If morphogen signals are directly mediated by chemical substance, where does it move? One of the possibilities is that chemical morphogens move in the extracellular space just beneath a cuticle cover. However, the dorsal surface is attached to the cuticle at least in the early pupal stages, and thus, extracellular space may not be liquidous. On the other hand, the haemolymph space between the two epithelia is also difficult to move through, because of the relatively intense haemolymph movement. Furthermore, the dynamic movement of the wing tissue demonstrated in this study likely makes extracellular “long-distance” delivery of morphogens very difficult because a stable gradient in the extracellular space, if formed, would be agitated. Nijhout (1991)^1^ has proposed that epithermal feet and gap junctions are the means for morphogen delivery to adjacent cells. We additionally propose that the morphogens may be passed to adjacent cells via vesicles and/or epidermal feet and to distant cells via cytonemes, which are cellular structures that were identified in *Drosophila melanogaster*^63–68^ as well as in butterflies^49,51^. However, chemical morphogens are considered to function in a final step in a relatively local range in the induction model^5^. We have proposed unconventional long-distance morphogenic signals, i.e., calcium waves^5,50^ and physical distortion waves^5,13^, that could work together with the conventional chemical morphogen. The earliest emergence of the discal spot detected in the present study may imply that the prospective discal spot could function as the source of non-chemical, long-distance morphogenic signals that coordinate wing-wide colour patterns in many nymphalid butterflies.

## Methods

### Butterflies

The following 9 nymphalid butterfly species were examined: the peacock pansy *Junonia almana* (Linnaeus, 1758) (Nymphalinae), the blue pansy *Junonia orithya* (Linnaeus, 1758) (Nymphalinae), the Indian red admiral *Vanessa indica* (Herbst, 1794) (Nymphalinae), the Indian fritillary *Argyreus hyperbius* (Linnaeus, 1763) (Heliconiinae), the staff sergeant *Athyma selenophora* (Kollar, [1844]) (Limenitidinae), the plain tiger *Danaus chrysippus* (Linnaeus, 1758) (Danainae), the common tiger *Danaus genutia* (Cramer, [1779]) (Danainae), the large tree nymph *Idea leuconoe* Erichson, 1834 (Danainae), and the Ceylon blue glassy tiger *Ideopsis similis* (Linnaeus, 1758) (Danainae). Additionally, 2 lycaenid butterfly species, *Zizeeria maha* (Kollar, [1844]) (Lycaeninae) and *Lycaena phlaeas* (Linnaeus, 1761) (Lycaeninae), and 1 pierid species, *Pieris rapae* (Linnaeus, 1758) (Pierinae), were examined.

We collected female adult individuals or larvae from Okinawa-jima Island or Ishigaki-jima Island, the Ryukyu Archipelago, Japan, except the 2 lycaenid species, which were collected in Osaka, Japan. However, *Z. maha* was collected both from Okinawa (*Z. maha okinawana*) and Osaka (*Z. maha argia*). Eggs were collected from the field-collected females. Larvae were fed their natural host plants at an ambient temperature of approximately 27 °C. No permissions were required to collect these butterflies from the wild, to rear this butterfly in the laboratory and to perform experiments with these butterflies in Okinawa, Japan.

### Butterfly colour patterns

The 9 nymphalid species used in the present study have various features that are suitable for examining different aspects of the colour pattern development. The two *Junonia* species were suitable for eyespot development and to simultaneously examine the relationship between elements and background because of their large eyespots on the dorsal forewings. In contrast to *Junonia* species, *V. indica* has small compromised eyespots (i.e., black spots) on the dorsal hindwing. Two species, *Argyreus hyperbius* and *Athyma selenophora*, have several small elements on the ventral forewing, making them suitable for examining the emergence order among elements of the same colour. The ventral forewings of *J. almana* and *V. indica* are also useful for examining the emergence order among elements of the same colour on the same wing surface. Among the 4 Danainae species used in this study, *D. chrysippus* and *D. genutia* had distinct black and red (orange) areas, which are suitable for observing interactions between them in the colour boundary. The latter also has distinct vein-dependent patterns. *Idea leuconoe* and *Ideopsis similis* are Danainae species but have very different colour patterns from *Danaus* species.

As a representative lycaenid species that has been used for developmental and evolutionary studies^69–72^, *Z. maha* was examined in the present study. On the ventral side, there are distinct black spots against the greyish background. Another lycaenid species examined was *L. phlaeas*, which has black spots both on the dorsal and ventral sides. Moreover, this species has reddish area along the hindwing margin. In addition, *P. rapae* was examined as a representative pierid species.

### Pupal operation

Pupae were subjected to the forewing-lift operation^18,21^, except those of some Danainae, Lycaenidae, and Pieridae species, at the ambient temperature. The right pupal forewing was lifted with forceps within 30 min post-pupation. The surface of both forewing and hindwing were then covered with a sheet of transparent plastic wrap. The image recording was started within 1 h post-pupation except *D. chrysippus* (Fig. 6b) (started approximately 173 h post-pupation), *Idea leuconoe* (Fig. 7a) (started approximately 4 h post-pupation), *Ideopsis similis* (Fig. 7c) (started approximately 6 h post-pupation), and *L. phlaeas* (Fig. 9c) (started approximately 39 h post-pupation). For most Danainae and some Lycaenidae and Pieridae pupae, the forewing-lift operation was not performed, and the dorsal side of the forewing was recorded through the transparent cuticle. Because the pupal cuticle of the Danainae species was too soft to withstand the recording process, the image recording was not started immediately after pupation; it was started within 1 d post-pupation or much later in some cases. Pupal body was fixed with double-sided adhesive tape at the bottom of a plastic dish, if necessary. In the forewing-lift configuration, the lifted forewing often died but the entire development of other parts of the body (including the hindwing) developed normally, and these individuals often eclosed. In some cases, the hindwing died but the forewing and other parts developed normally. In these cases, a living wing was considered to have undergone normal development.

### Wing imaging systems

The whole-wing images were taken using a Canon EOS digital single-lens reflex camera (Tokyo, Japan). The camera was controlled with Canon EOS Utility software (Tokyo, Japan). The software was installed in a PC with the Windows Operating System. Static images were taken every 1 min. One of the following 4 imaging systems were used. (1) Canon EOS 40D, Canon macro lens (EF-S 60 mm F2.8 macro USM) (Tokyo, Japan), ISO800, a shutter speed of 1/5 sec, and a diaphragm setting of F10 for *J. almana* and *V. indica*. (2) Canon EOS Kiss X2, Canon normal lens (EF-S 18–55 mm F3.5–5.6 IS), together with Kenko lens filter MC close-up lens No.10 58 mm (Tokyo, Japan) and Canon close-up lens 500D 58 mm, ISO1600, a shutter speed of 1/200 sec (or 1/160 sec for *P. rapae*), and a diaphragm setting of F14 for *Argyreus hyperbius*, *Athyma selenophora*, *Z. maha* (Fig. 8b), and *P. rapae* (Fig. 10a). (3) Canon EOS Kiss X7, Canon normal lens (EF-S 18–55 mm F3.5–5.6 IS STM), together with Kenko lens filter MC close-up lens No.10 58 mm (Tokyo, Japan) and Canon close-up lens 500D 58 mm, ISO1600, a shutter speed of 1/200 sec, and a diaphragm setting of F14 for Danainae species and *P. rapae* (Fig. 10b). (4) Canon EOS Kiss X7, Canon normal lens (EF-S 18–55 mm F3.5–5.6 IS STM), together with Kenko lens accessory reverse adaptor 58 mm, (Tokyo, Japan), ISO1600, a shutter speed of 1/200 sec, and a diaphragm setting of maximum aperture for *Z. maha* (Fig. 8a,c) and *L. phlaeas*. Exceptions were images of *J. orithya* (see below).

The images were then compiled into a time-lapse mp4 movie using software Windows Movie Maker version 2012 (Microsoft). Frame rate per second was 29.97 fps with 720 pixel (width) × 480 pixel (height) in all movies (Supplementary Videos S1–S19). Movie properties were checked with Mediainfo 19.03.1 (MediaArea.net SARL). The panel numbers in figures show time points in min after the beginning of image recording.

Images of *J. orithya* were obtained every 2 min using a Keyence VHX-1000 digital microscope (Osaka, Japan)^48^. Some images of *J. orithya* have already been published in Iwata *et al*.^48^. In the present study, however, an image analysis for these data was further performed, and non-redundant images and their analysis results, which have not been published elsewhere, are presented in Fig. 3.

## Electronic supplementary material

Supplementary Information

Supplementary Video S1

Supplementary Video S2

Supplementary Video S3

Supplementary Video S4

Supplementary Video S5

Supplementary Video S6

Supplementary Video S7

Supplementary Video S8

Supplementary Video S9

Supplementary Video S10

Supplementary Video S11

Supplementary Video S12

Supplementary Video S13

Supplementary Video S14

Supplementary Video S15

Supplementary Video S16

Supplementary Video S17

Supplementary Video S18

Supplementary Video S19

## Data Availability

Almost all data generated or analysed during this study are included in this published article and its Supplementary Information files. The datasets not included in this article and its Supplementary Information files are available from the corresponding author on reasonable request.
