# Supplementary material for: Developmental dynamics of butterfly wings: real-time in vivo whole-wing imaging of twelve butterfly species
Source: Sci Rep. 2018 Nov 15;8:16848. doi: 10.1038/s41598-018-34990-8 (PMC6237780; doi:10.1038/s41598-018-34990-8)
Supplement: Supplementary file 1 — Supplementary Information [file 41598_2018_34990_MOESM1_ESM.pdf]

Scientific Reports

Supplementary Information

Developmental dynamics of butterfly wings: real-time *in vivo* whole-wing imaging of twelve butterfly species

Masaki Iwata, Motosuke Tsutsumi & Joji M. Otaki

The BCPH Unit of Molecular Physiology, Department of Chemistry, Biology and Marine Science, University of the Ryukyus, Okinawa 903-0213, Japan

Correspondence and requests for materials should be addressed to J.M.O. (otaki@sci.u-ryukyu.ac.jp)

Supplementary Videos S1-S19

Note: All video files were 720 pixel (width) × 480 pixel (height) at 29.97 fps. These are original files that are not edited except telop insertion for identification and file size adjustments.

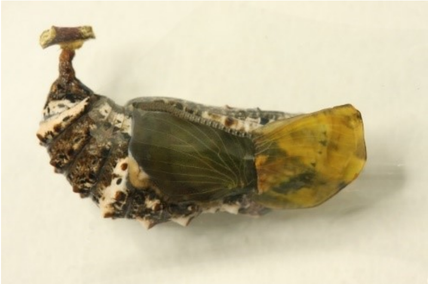

Supplementary Video S1. Developmental time course of the pupal forewing and hindwing of *J. almana*. This video corresponds to Fig. 1a (the ventral forewing and the dorsal hindwing) and Fig. 2 (the ventral forewing).

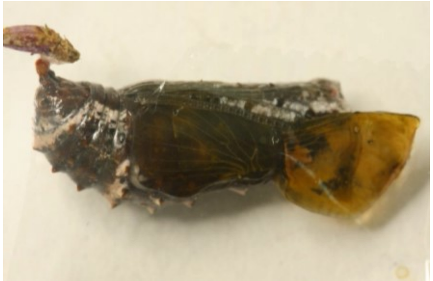

Supplementary Video S2. Developmental time course of the pupal hindwing of *J. almana*. This video corresponds to Fig. 1b (the dorsal hindwing). This individual is different from that of Supplementary Video S1 (Fig. 1a).

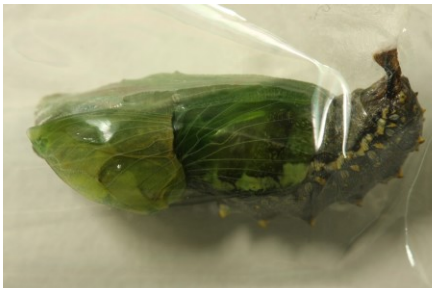

Supplementary Video S3. Developmental time course of the pupal forewing and hindwing of *V. indica*. This video corresponds to Fig. 4a (the ventral forewing and the dorsal hindwing) and Fig. 4b (the dorsal hindwing).

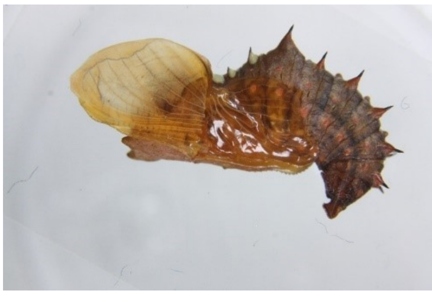

Supplementary Video S4. Developmental time course of the pupal forewing of *Argyreus hyperbius*. This video corresponds to Fig. 5a (the ventral forewing).

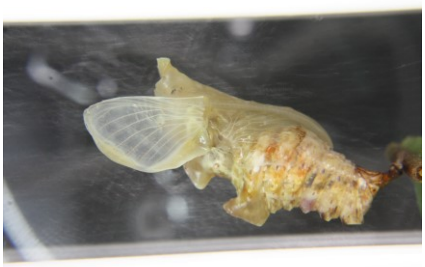

Supplementary Video S5. Developmental time course of the pupal forewing of *Athyma selenophora*. This video corresponds to Fig. 5b (the ventral forewing).

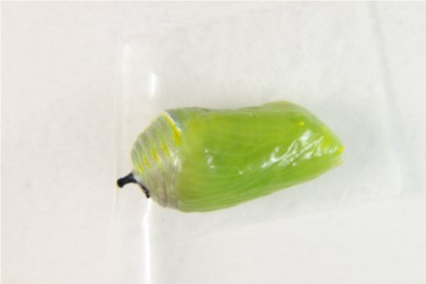

Supplementary Video S6. Developmental time course of the pupal forewing of *D. chrysippus*. This video corresponds to Fig. 6a (the dorsal forewing).

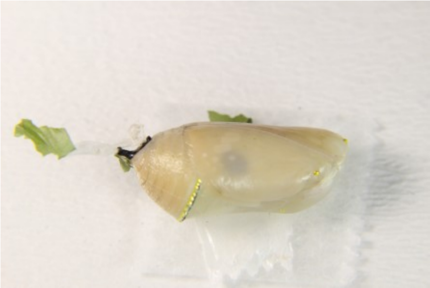

Supplementary Video S7. Developmental time course of the pupal forewing of *D. chrysippus*. This video corresponds to Fig. 6b (the dorsal forewing). This individual is different from that of Supplementary Video S6 (Fig. 6a). Only the coloration stage is recorded in this video.

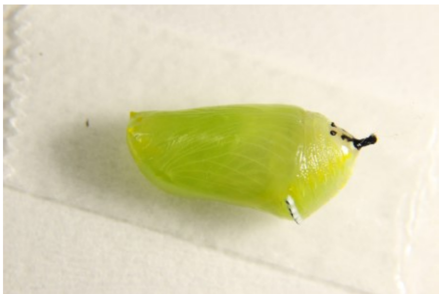

Supplementary Video S8. Developmental time course of the pupal forewing of *D. genutia*. This video corresponds to Fig. 6c (the dorsal forewing).

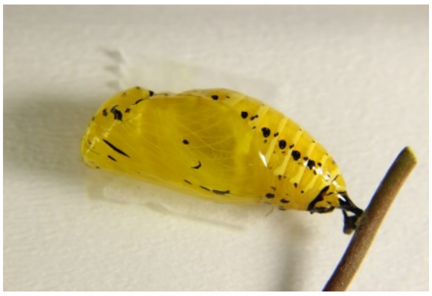

Supplementary Video S9. Developmental time course of the pupal forewing of *Idea leuconoe*. This video corresponds to Fig. 7a (the dorsal forewing).

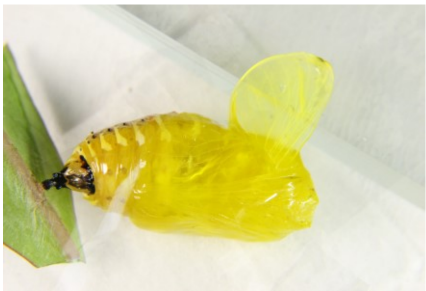

Supplementary Video S10. Developmental time course of the pupal hindwing of *Idea leuconoe*. This video corresponds to Fig. 7b (the dorsal hindwing).

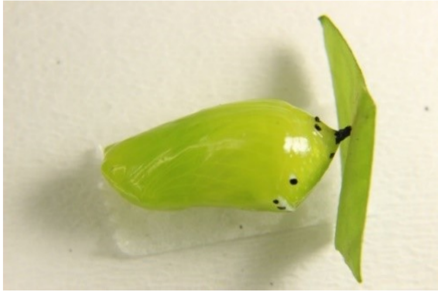

Supplementary Video S11. Developmental time course of the pupal forewing of *Ideopsis similis*. This video corresponds to Fig. 7c (the dorsal forewing).

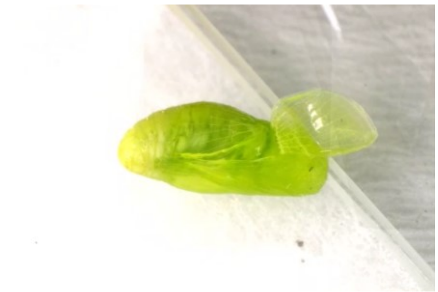

Supplementary Video S12. Developmental time course of the pupal forewing and hindwing of *Zizeeria maha*. This video corresponds to Fig. 8a (the ventral forewing and the dorsal hindwing).

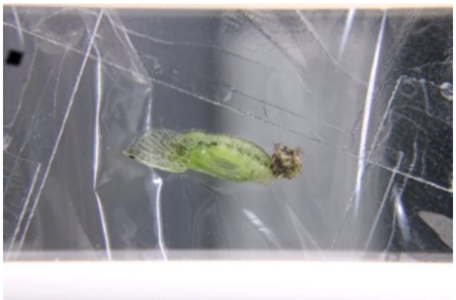

Supplementary Video S13. Developmental time course of the pupal forewing and hindwing of *Zizeeria maha*. This video corresponds to Fig. 8b (the ventral forewing).

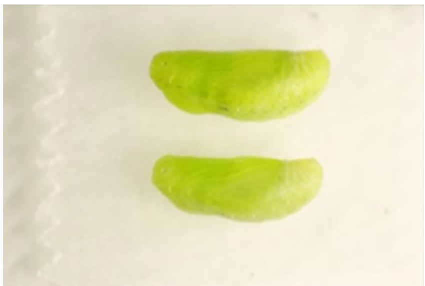

Supplementary Video S14. Developmental time course of the pupal forewing of *Zizeeria maha*. This video corresponds to Fig. 8c (the dorsal forewing). The individual at the top developed to the end, whereas the individual at the bottom died soon after the imaging.

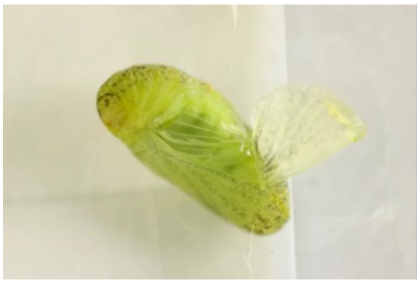

Supplementary Video S15. Developmental time course of the pupal forewing and hindwing of *Lycaena phlaeas*. This video corresponds to Fig. 9a (the ventral forewing and the dorsal hindwing).

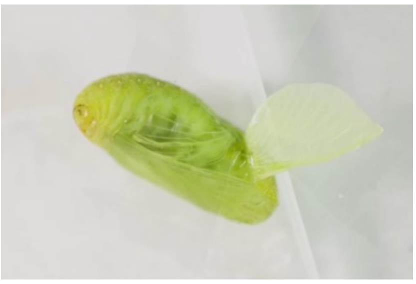

Supplementary Video S16. Developmental time course of the pupal hindwing of *Lycaena phlaeas*. This video corresponds to Fig. 9b (the dorsal hindwing).

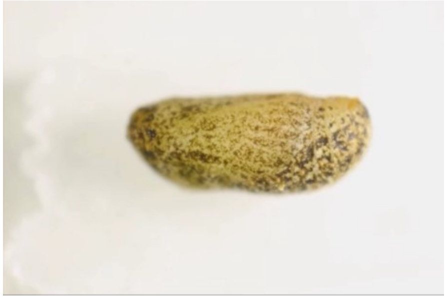

Supplementary Video S17. Developmental time course of the pupal forewing of *Lycaena phlaeas*. This video corresponds to Fig. 9c (the dorsal forewing).

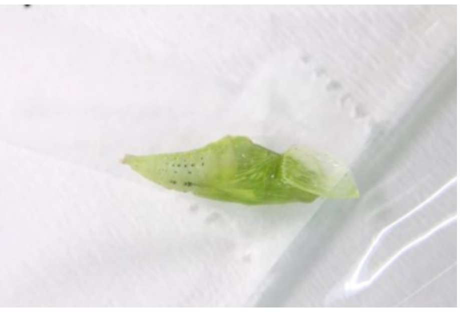

Supplementary Video S18. Developmental time course of the pupal forewing and hindwing of *Pieris rapae*. This video corresponds to Fig. 10a (the ventral forewing and the dorsal hindwing).

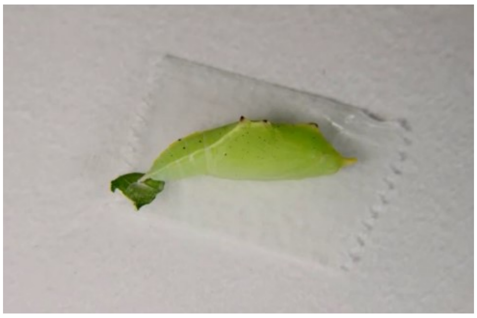

Supplementary Video S19. Developmental time course of the pupal forewing of *Pieris rapae*. This video corresponds to Fig. 10b (the dorsal forewing).
